# The Changed Route of Anterior Tibial Artery due to Healed Fracture

**DOI:** 10.1155/2016/5013013

**Published:** 2016-02-25

**Authors:** Kemal Gökkuş, Ergin Sagtas, Nuri Comert, Mehmet Bekir Unal, Murat Baloglu

**Affiliations:** ^1^Orthopaedics and Trauma Department, Antalya Memorial Hospital, 07025 Antalya, Turkey; ^2^Radiodiagnostic Department, Antalya Memorial Hospital, 07025 Antalya, Turkey; ^3^Cardiology Department, Antalya Memorial Hospital, 07025 Antalya, Turkey; ^4^Orthopaedics and Trauma Department, Goztepe Medical Park Hospital, Istanbul, Turkey

## Abstract

We would like to highlight unusual sequelae of healed distal third diaphyseal tibia fracture that was treated conservatively 36 years ago, in which we incidentally detected peripheral CT angiography. The anterior tibial artery was enveloped three-quarterly by the healing callus of the bone (distal tibia).

## 1. Introduction 

In the distal third tibial fractures, the surgeon has multiple options: conservative treatment, intramedullary nailing, open reduction and plate and screw fixation, and minimally invasive plate osteosynthesis (MIPO) technique [1–3].

In this report we would like to highlight unusual sequelae of healed distal third diaphyseal tibia fracture that was treated conservatively 36 years ago, in which we incidentally detected peripheral CT angiography.

## 2. Case 

A 67-year-old heavy smoker male patient with a history of atherosclerotic heart disease, hypertension, Type II DM, and hyperlipidemia came to our cardiology department and was treated with the following drug regimen: Insulin (25 IU/sec), Metformin (1 gr *∗* 2/day), Glimepiride (4 mg/day), Nitrates (50 mg/day), Irbesartan (300 mg/day), and Simvastatin (40 mg/day).

The patient was referred to our cardiologist for intermittent claudication. Following routine tests, the cardiologist decided to perform a peripheral CT angiography. The CT revealed an occlusion on right superficial femoral artery (right iliopopliteal bypass surgery was scheduled for following week).

During our radiologist's CT angiogram assessment, he noticed an unusual course of left tibia anterior artery. The anterior tibial artery was enveloped* three-quarterly by the healing callus of the bone* (distal tibia).

**Figure 1:**
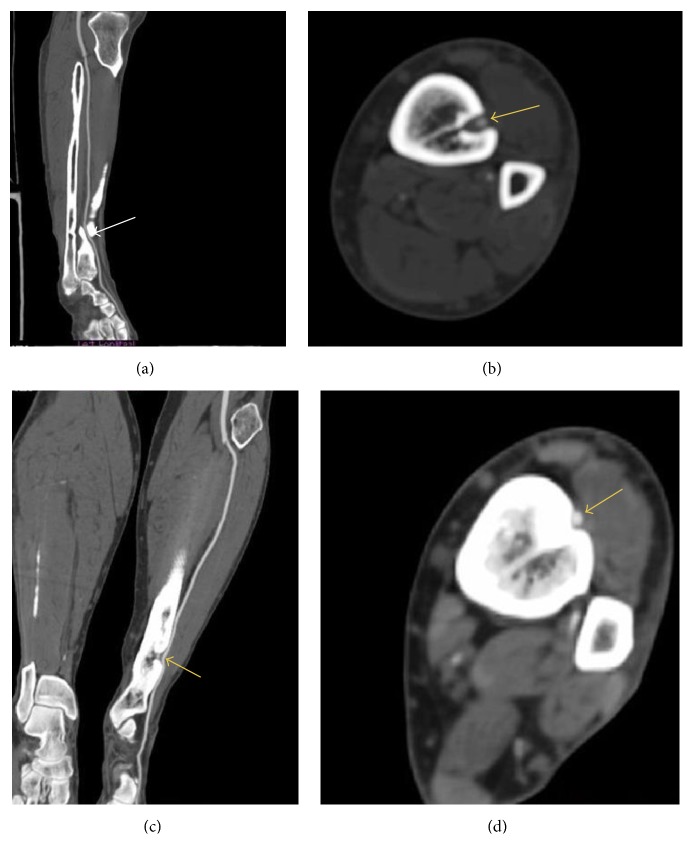
(a) Notice the anterior tibial artery courses in the tibial bone (white arrow). (b) Transverse section: notice that artery made its own tunnel in the tibial cortex (yellow arrow). (c) The appearance of distal tibia very suggestive about the healed fracture. Notice the tibial artery course (yellow arrow). (d) Tibialis anterior artery courses on the recess (yellow arrow).

The anterior tibial artery coursed in tibial cortex made its own pathway within the bone and returned to its original anatomic course (see Figure 1). Noticing this, the patient was asked about tibial fractures he had in the past. Patient reported a distal 1/3 tibial diaphyseal fracture that happened 36 years ago. The fracture was originally treated using a complete leg cast (nonsurgical treatment) by orthopedic surgeons and fracture unification was uneventful. The patient had a normal life course.

During the examination of the fracture done by orthopedists 36 years ago, they did not detect a problem about circulation of the area and distal pulses were also normal. Even though we requested the previous X-rays of the patient, the patient was unable to obtain them since the original hospital was since then moved 3 times and most of its archives were either lost or destroyed.

## 3. Discussion

Remembering the anatomy of the anterior tibial artery, the anterior tibial artery is the smallest of the terminal branches of the popliteal artery. It arises at the level of the lower border of the popliteus muscle and passes forward into the anterior compartment of the leg through an opening in the upper part of the interosseous membrane. It descends on the anterior surface of the interosseous membrane, accompanied by the deep peroneal nerve. In the upper part of its course, it lies deep beneath the muscles of the compartment. The artery then descends between the tibialis anterior and extensor digitorum longus muscles [4, 5].

In our case the anterior tibial artery coursed in tibial cortex made its own pathway within the bone and returned to its original anatomic course (see Figure 1).

Today, these types of fractures can be treated using either intramedullary nails or MIPO (Minimal Invasive Plate Osteosynthesis). They allow the surgeon to perform fracture reduction and a secure fixation without a direct view of the fracture. However, until the end of 1970s era, treatment approach was generally towards using nonsurgical methods. The novel usage of CT angiography after 1990s era also explains this treatment choice. However, today CT angiography is used widely in most developed countries in diagnosis. However, under those circumstances where peripheral CT angiography was not available, if the surgeons decide to make closed reduction with intramedullary nailing instead of conservative treatment (cast immobilisation), the surgeons probably would live nightmare about the massive bleeding due to hardware puncture to artery.

Pseudoaneurysm after tibial nailing was also reported in the literature by Han et al. [6].

In this case, both the patient and the orthopedic surgeons were quite lucky not to come across such a major complication.

As seen in both Figures 1(a) and 1(c), the rugged view of tibia can be interpreted as a previously unified fracture and shows us that this is not a rare congenital defect or an anatomic variant.

Also we scanned literature and could not find such a variation regarding the unusual course of anterior tibial artery on this pathway. Anterior tibial artery variations and abnormalities are mostly located in proximal part in which the vessel originated from the popliteal artery root [7–9].

The condition of hypoplastic anterior tibial artery or variation, associated with continuation of fibular (peroneal) artery as dorsalis pedis artery, was also reported by Shetty et al. [10].

Those images carry a high educational value since it is extremely rare to come across such rare cases with such detailed imaging studies. In our literature scan on anterior tibial artery entrapment in distal third tibial fracture, only one article was found in this subject, which proves our point [11].

## 4. Conclusion

One must keep in mind that not all tibial fractures are uneventful and complication-free that they might always cause a vascular injury, even in times when distal pulses were recorded as normal.
